# Transcriptional hallmarks of cancer cell lines reveal an emerging role of branched chain amino acid catabolism

**DOI:** 10.1038/s41598-017-08329-8

**Published:** 2017-08-10

**Authors:** Ieva Antanavičiūtė, Valeryia Mikalayeva, Ieva Ceslevičienė, Gintarė Milašiūtė, Vytenis Arvydas Skeberdis, Sergio Bordel

**Affiliations:** 1 Institute of Cardiology, Lithuanian University of Health Sciences, 15 Sukilėlių Ave., LT- 50162 Kaunas, Lithuania; 2 Department of Biology and Biological Engineering, Chalmers University of Technology, Kemivägen 10, SE-41296 Göteborg, Sweden

## Abstract

A comparative analysis between cancer cell lines and healthy dividing cells was performed using data (289 microarrays and 50 RNA-seq samples) from 100 different cancer cell lines and 6 types of healthy stem cells. The analysis revealed two large-scale transcriptional events that characterize cancer cell lines. The first event was a large-scale up-regulation pattern associated to epithelial-mesenchymal transition, putatively driven by the interplay of the SP1 transcription factor and the canonical Wnt signaling pathway; the second event was the failure to overexpress a diverse set of genes coding membrane and extracellular proteins. This failure is putatively caused by a lack of activity of the AP-1 complex. It was also shown that the epithelial-mesenchymal transition was associated with the up-regulation of 5 enzymes involved in the degradation of branched chain amino acids. The suitability of silencing one of this enzymes (branched chain amino acid transaminase 2; BCAT2) with therapeutic effects was tested experimentally on the breast cancer cell line MCF-7 and primary cell culture of breast tumor (BCC), leading to lower cell proliferation. The silencing of BCAT2 did not have any significant effect on ASM and MCF10A cells, which were used as models of healthy dividing cells.

## Introduction

Side effects are among the main problems related to chemotherapy. Most of the drugs used in chemotherapy target DNA replication or key regulators of the cell cycle^1^, which has a negative impact not only on malignant cells but also on healthy proliferating cells (stem cells and progenitors), leading to stem cell depletion and impaired renewal and function of healthy tissues^2^. Therefore, identifying systematic differences between cancer cells and healthy dividing cells, is fundamental to identify therapeutic windows that could be exploited to target cancer cells while minimizing side effects. The development of high throughput omics technologies such as cDNA microarrays and more recently, RNA-sequencing, has led to the accumulation of large datasets that constitute rich sources of information allowing us to identify systematic differences that characterize cancer cells. These transcriptional differences are expected to provide keys for the design of therapies targeting cancer cells specifically without damaging healthy dividing cells and therefore to minimize the secondary effects associated with stem cell depletion caused by chemotherapy.

Cancer cell lines have their origin in healthy stem cells or progenitors^3, 4^ that undergo a series of mutations resulting in a tumorigenic phenotype. Recent high-throughput sequencing studies of human cancers^5–7^ have revealed hundreds of somatic mutations associated with cancer; however, very few genes were found to be mutated in large fractions of the studied samples. In each cancer type, only about 4 genes were altered in more than 20% of the studied samples^8^. The TP53 tumor suppressor is the most frequently mutated gene, but it is still far from being present in all sequenced cancers. Despite this large heterogeneity in the mutations that trigger malignant transformations, cancer has been characterized in terms of a small set of hallmarks described by Hannahan and Weinberg^9^. The acquisition of these hallmarks is likely to be associated with well-coordinated large-scale transcriptional changes that are absent in healthy cells (healthy stem cells and progenitors in particular). Here we have analyzed a large set of gene expression data (microarrays and RNA-seq) from cancer cell lines and healthy proliferating cells, with the aim of identifying transcriptional hallmarks present in cancer cell lines and absent in healthy cells.

## Results

### Transcriptional hallmarks of cancer cell lines

**Figure 1:**
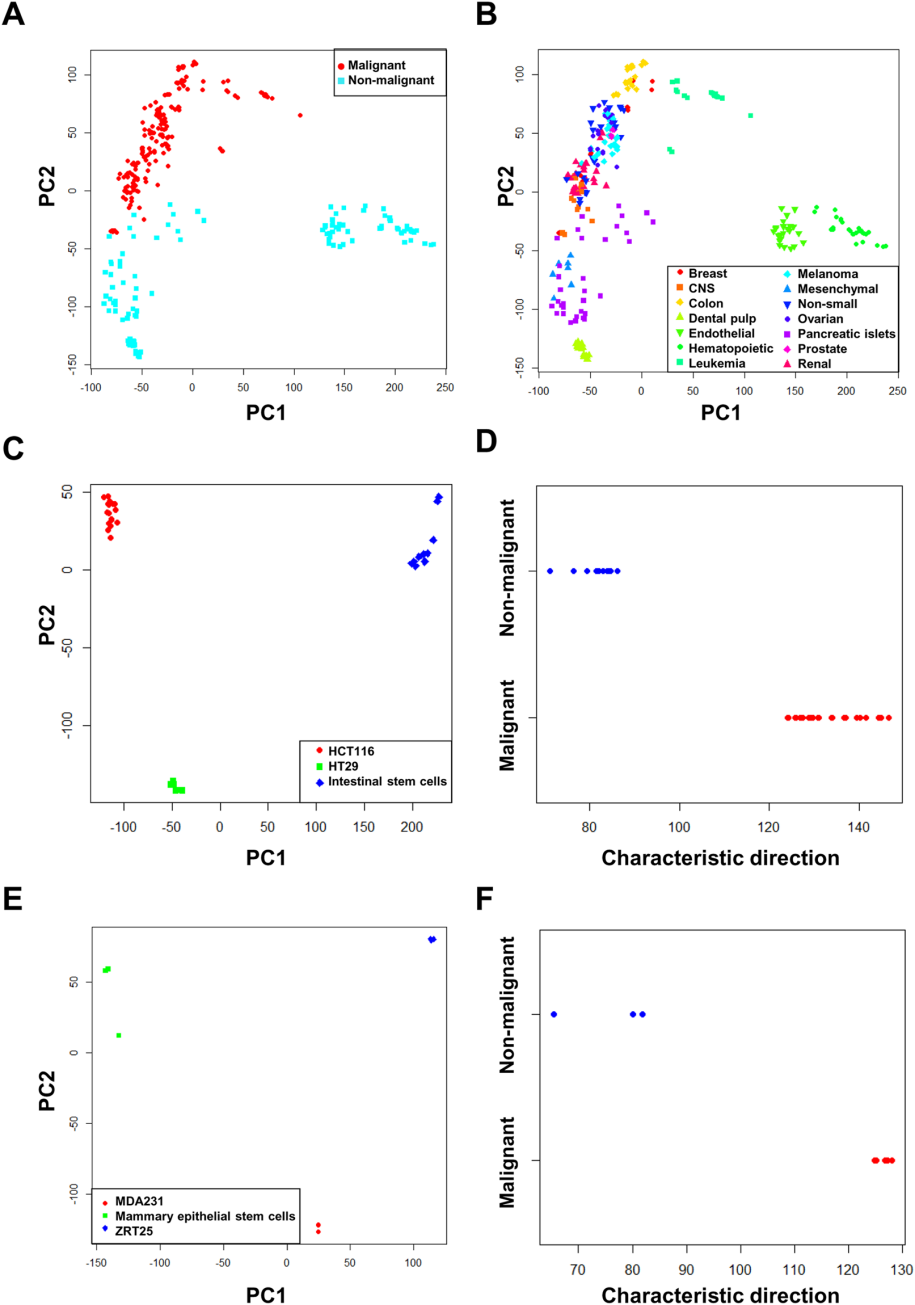
Exploratory analysis of gene expression profiles. The second principal component appears to separate CICs from healthy dividing cells (**A**). The first principal component seems to separate the cells with an origin in the hematopoietic system: hematopoietic stem cells, endothelial progenitor cells, and leukemia (**B**). The colon cancer cell lines HCT116 and HT29 differ strongly between each other and with respect to intestinal stem cells (**C**); however, the projection of their expression profiles along the characteristic direction clusters both cancer cell lines together and separates them from the stem cells (**D**). The same is observed for the breast cancer cell lines MDA231 and ZRT75 (**E**) when compared to mammary epithelial stem cells (**F**).

In order to identify the transcriptional changes that make cancer cell lines different from healthy dividing cells, we analyzed 289 microarrays from the GEO database (the accession numbers are reported in Supplementary Table S1). These microarrays correspond to the cancer cell lines of the NCI-60 collection and 5 types of healthy dividing cells that include: beta cells from pancreatic islets, hematopoietic stem cells, dental pulp stem cells, endothelial progenitor cells, and mesenchymal stem cells. After microarray normalization, a principal component analysis was performed in order to visualize the structure of the data (Fig. 1A,B). It appears that the first principal component discriminates between cells with their origin in the hematopoietic system (hematopoietic stem cells, endothelial progenitor cells and leukemia cell lines) from the rest; however, leukemia cells are strongly displaced toward the proximity of the other cancer cell lines. The second principal component appears to discriminate cancer cell lines from healthy dividing cells. This suggests that it is indeed possible to find a distinct transcriptional pattern that characterizes cancer cell lines.

We used a linear discrimination analysis^10^ in order to identify the plane in the gene expression space that best separates cancer cell lines from healthy dividing cells. The vector perpendicular to this plane is described as the characteristic direction (see supplementary material). By projecting the expression profile of each sample on the characteristic direction, we can fully discriminate tumorigenic cells from healthy dividing cells (Supplementary Fig. S1). The genes with statistically significant contributions (negative and positive) to the characteristic direction (with false discovery rates lower than 0.05) are reported in Supplementary File SF2.

In order to assess if the identified expression profile is a general characteristic of tumorigenic cells or just an artifact of our choice of samples, we tested two datasets, each of which contained a stem cell type different from those previously used (colon stem cells and mammary epithelial stem cells) and two different cancer cell lines originated from the same tissue (colon and breast cancer cell lines). The GEO accession numbers and cell lines are reported in the supplementary material (Table S2). Each validation dataset was normalized individually (independently from each other and from the initial microarray set). After normalization, we performed a principal component analysis showing that the cancer cell lines differ from each other in terms of expression as much as they differ from the stem cells of their tissue of origin. However, if we project their expression profiles on the characteristic direction (which was obtained independently using different cell lines), both cancer cell lines get clustered together and clearly separated from the stem cells (Fig. 1C–F). These results suggest that the expression pattern described by the obtained characteristic direction is a general feature that discriminates cancer cell lines from healthy dividing cells.

In order to understand the mechanisms behind the coordinated changes in expression in the identified gene sets, we analyzed the list of genes with significant contributions to the characteristic direction (0.05 false discovery rates) using GeneCodis^11–13^ for transcription factor enrichment. The top scoring transcription factors were LEF1 (with p-values of 9.3 e-47 for down-regulated genes and 3.33 e-24 for up-regulated genes), NFAT (p-value of 4.87 e-45 for down-regulated genes), SP1 (p-value of 1.68 e-40 for up-regulated genes), and FOXO4 (p-values of 5.56 e-44 for down-regulated genes and 1.61 e-19 for up-regulated genes). The differentially expressed genes regulated by each of these transcription factors are reported in Supplementary File SF2.

### An up-regulation pattern associated with epithelial-mesenchymal transition

The transcription factor coded by LEF1 interacts with β-catenin in the last step of the Wnt signaling pathway. This interaction induces the expression of several key regulators of the cell cycle and results in symmetric division^14, 15^. Mutations resulting in the constitutive activation of the Wnt/β-catenin pathway have been reported in many cancer types^16^. It has also been shown that the activation of LEF1 is enough to induce neoplastic transformation in chicken embryo fibroblasts^17^.

FOXO proteins have been reported to bind β-catenin^18^, and the transcription level of the FOXO targets has been shown to increase when β-catenin binds the FOXO transcription factors^19^. Therefore, the transcriptional changes mediated by LEF1 and FOXO4 could have a common origin in the Wnt/β-catenin pathway.

SP1 is involved in the epithelial-mesenchymal transition^20^, and it is necessary to confer metastatic capabilities to tumorigenic cells. Inhibition of SP1 has been shown to have antitumor effects^21^. The results from the enrichment analysis performed with GeneCodis showed that the SP1 gene is among those up-regulated in cancer cell lines and controlled by FOXO4, together with other genes involved in the TGF-β pathway (see Supplementary Files SF2 and SF3). On the other hand, 9 genes (see Supplementary File SF3) belonging to the Wnt signaling pathway are up-regulated in cancer cell lines and controlled by SP1 (according to GeneCodis). This suggests the existence of a positive feedback involving SP1, the Wnt pathway, and FOXO4, resulting in a large-scale transcriptional change. SP1 up-regulation has been recently shown experimentally to activate Wnt/β-catenin signaling in MCF-7 and MCF-10A cells^22^, which is consistent with our observations. Inactivation of the Wnt antagonist WTX has been shown to induce β-catenin activation and result in the accumulation of mesenchymal precursor cells in mice^23^, which also suggests that β-catenin is indeed involved in epithelial-mesenchymal transition.

**Figure 2:**
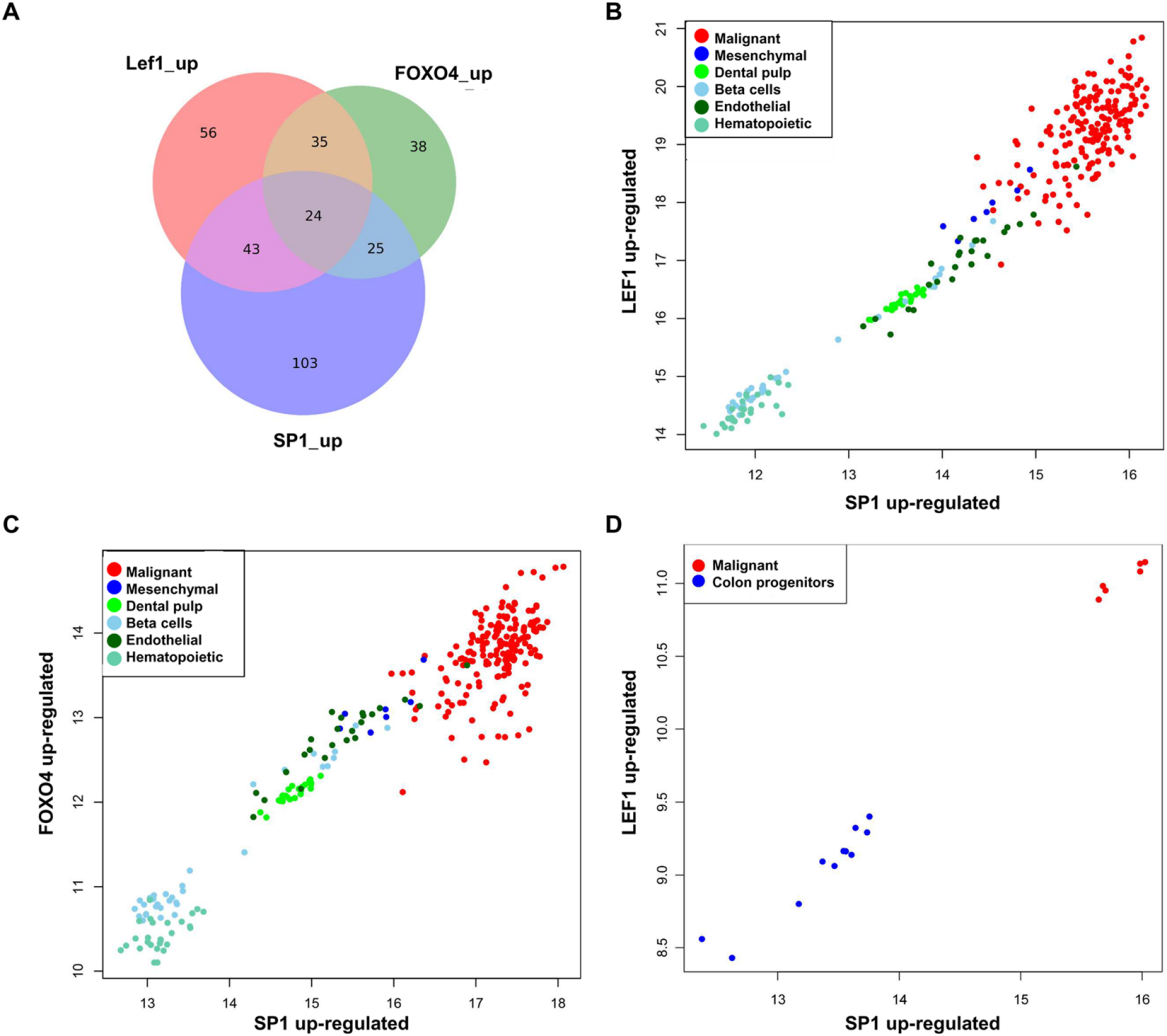
Expression of the SP1, FOXO4, and LEF1 regulated genes. Overlap between genes up-regulated in cancer cell lines and regulated by each of the three transcription factors (according to GeneCodis) (**A**). Correlation between the aggregated expressions of SP1 up-regulated genes and LEF1 up-regulated genes (**B**). Correlation between the aggregated expressions of SP1 up-regulated genes and FOXO4 up-regulated genes (**C**). Correlation between the aggregated expressions of SP1 up-regulated genes and LEF1 up-regulated genes in colon stem cells and the colon cancer cell line HT29 (**D**).

The genes identified as up-regulated in cancer cell lines (using the method described in the section Statistical Methods in the supplementary material) and regulated by LEF1, FOXO4, and SP1, respectively (according to GeneCodis^11–13^), are reported in Supplementary File SF2. Among these genes, 197 are regulated by just one of the three transcription factors, while 127 are regulated by two or three (see Venn diagram in Fig. 2A).

The characteristic direction was projected (as described in the supplementary material) on the set of 158 genes putatively regulated by LEF1. This projection was used as an estimation of the activity of the LEF1 transcription factor in each cell line. The characteristic direction was also projected on the set of 128 genes putatively regulated by SP1 excluding the 67 genes that are regulated by SP1 and LEF1. These genes were excluded in order to compare gene sets that do not overlap and to avoid finding spurious correlations. A Pearson correlation coefficient of 0.968 was obtained between the projections on each of the gene sets (Fig. 2B). This confirms that the activities of LEF1 and SP1 are correlated.

The same procedure was repeated using the set of 122 genes putatively regulated by FOXO4 and 146 genes putatively regulated by SP1 and not by FOXO4 (Fig. 2C). In this case, a Pearson correlation coefficient of 0.958 was obtained between the projections on both gene sets, suggesting the existence of correlation between the activities of FOXO4 and SP1.

The cancer cell lines appear in the upper right corner of the correlation plots (Fig. 2B,C) closely situated to the mesenchymal stem cells, which suggests that the transcriptional pattern involving the coordinated up-regulation of genes controlled by SP1, LEF1, and FOXO4 corresponds to the phenomenon of epithelial-mesenchymal transition (SP1 is known to be involved in epithelial-mesenchymal transition^20^). The same pattern is observed on colon stem cells and colon cancer cells HT29, which were not used to compute the characteristic direction and which were normalized independently from the previous dataset (Fig. 2D). This confirms that the observed phenomenon is not restricted to the dataset of 289 microarrays that we used initially.

### A down-regulation pattern associated with loss of intercellular communication and attachment to the extra-cellular matrix

**Figure 3:**
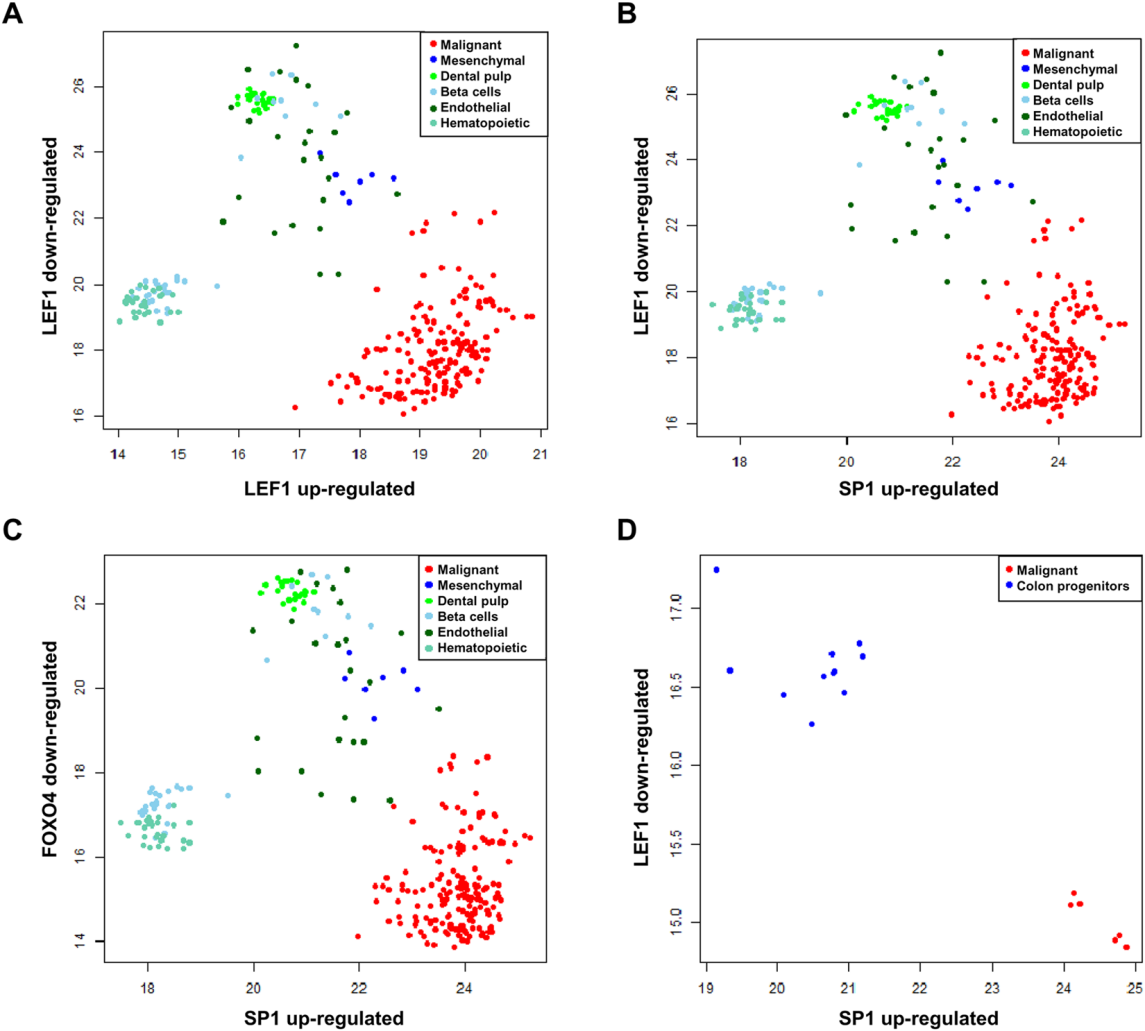
Projections of the characteristic direction on the LEF1 and SP1 regulons. Tumorigenic cells show a consistently higher LEF1 activity, while SP1 activity differentiates epithelial and mesenchymal cells.

The enrichment analysis revealed not only sets of up-regulated genes putatively controlled by LEF1 and FOXO4, but also large sets of down-regulated genes under the putative control of the same two transcription factors. In order to visualize the expression levels of these down-regulated gene sets in different cell lines, we started by plotting the projection of the characteristic direction (see supplementary material) on the genes down-regulated and controlled by LEF1 (noted as LEF1 down-regulated) versus the projection of the characteristic direction on the genes up-regulated and controlled by LEF1 (noted as LEF1 up-regulated) (Fig. 3A). In this case, we observe that the global expression of the down-regulated genes controlled by LEF1 actually increases in mesenchymal stem cells compared to hematopoietic stem cells and a part of the beta cells. The same pattern appears if we plot the global expression of the down-regulated genes controlled by LEF1 and FOXO4 versus the up-regulated genes controlled by SP1 (Fig. 3B,C). This phenomenon is equally observed in the dataset of colon stem cells and colon cancer cells HT29 (Fig. 3D).

The mentioned observations suggest that both sets of genes (LEF1 down-regulated and FOXO4 down-regulated) are actually up-regulated in normal epithelial-mesenchymal transitions, while their expression remains unmodified or decreases in cancer cell lines. Under this interpretation, cancer cell lines would be the result of incomplete epithelial-mesenchymal transition in which large sets of genes that are normally up-regulated, fail to get overexpressed.

**Figure 4:**
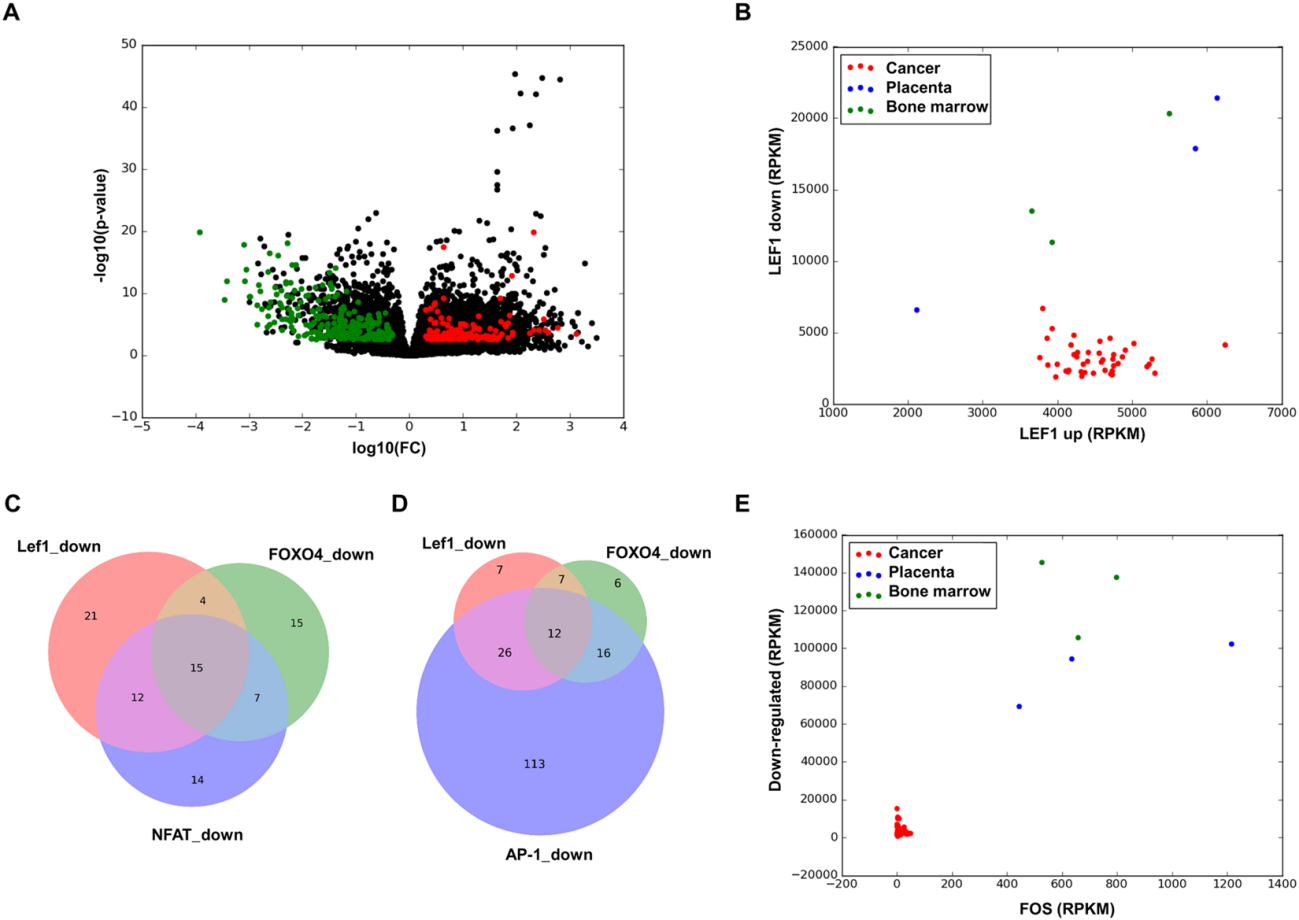
Volcano plot resulting from the differential expression analysis between cancer cell lines and mesenchymal stem cells. Genes found also to be differentially expressed in the microarray analysis are shown in red (up-regulated) and green (down-regulated) (**A**). Plot showing the sums of expression levels of genes regulated by LEF1 (**B**). Overlap between the consensus down-regulated genes controlled by LEF1, FOXO4, and NFAT (**C**). Overlap between the consensus down-regulated genes controlled by LEF1, FOXO4, and AP-1 (**D**). Plot showing the sum of expressions of all the down-regulated consensus genes versus the FOS gene (**E**).

The mentioned hypothesis was tested using a different technology to quantify gene expression. RNA-seq data of 44 cancer cell lines from the Human Proteome Atlas (BioProject accession number PRJNA183192) and 6 primary cultures of mesenchymal stem cells (3 from placenta and 3 from bone marrow)^24^ were compared. The raw sequence *fastq* files for mesenchymal stem cells were analyzed as described in the methods and a differential expression analysis was carried out in order to identify genes differentially expressed between cancer cell lines and healthy mesenchymal stem cells. The results are shown in the form of a *volcano* plot (Fig. 4A). Among the 1120 genes that appeared to have lower expression in cancer cell lines (based on the previously described microarray analysis), 260 were also down-regulated in the RNA-seq dataset (green points in Fig. 4A. These genes are referred to as consensus down-regulated genes from now on). This is 2.27 times higher than the expected number for a random set of 1120 genes and the p-value of a Fisher exact test is 1.88e-25. In contrast, only 135 of the 977 genes overexpressed in cancer cells were found to be overexpressed in the RNA-seq data (red points in Fig. 4A, further referred to as consensus up-regulated genes), which is only 0.8 times the value expected for a random dataset (statistically significant underrepresentation with a p-value of 0.017). This shows that genes up-regulated in cancer cell lines tend not to be also highly expressed in mesenchymal stem cells. On the other hand, genes with lower expression in cancer cell lines have higher expression in normal mesenchymal stem cells. The sum of the expression levels (measured in RPKM) of all the LEF1 up-regulated genes was plotted versus the sum of expressions of the LEF1 down-regulated genes (Fig. 4B). Remarkably, the same phenomenon as in Fig. 3A is observed. The LEF1 up-regulated genes show comparable levels of expression in cancer cell lines and mesenchymal stem cells, while the LEF1 down-regulated genes show lower expression levels in the cancer cell lines. A significant correlation of 0.72 (Pearson correlation coefficient) is observed between the sum of expressions of LEF1 up-regulated genes and SP1 up-regulated genes (data not shown), which is in agreement with the previously hypothesized relation between these transcription factors and epithelial-mesenchymal transition.

The reason for the observed failure to overexpress a large number of LEF1 and FOXO4 regulated genes in cancer cell lines could be partially explained by the NFAT transcription factor, which also appeared in the enrichment analysis of down-regulated genes and has been reported to be a Wnt signaling suppressor^25^. This was tested by comparing the overlap between genes regulated by LEF1, FOXO4, and NFAT within the set of consensus down-regulated genes. More than half of the LEF1 and FOXO4 down-regulated genes are not regulated by NFAT (Fig. 4C), which suggests that there should be an extra explanation for their down-regulation.

Two different approaches were used to identify putative transcription factors controlling the expression of the consensus down-regulated genes. Firstly, we performed a new enrichment analysis of the consensus down-regulated genes, this time using DAVID 6.7^26–28^. The top enriched binding motifs corresponded to the transcription factors BACH2 (p-value 6.5e-13), FOXO1 (7.3e-12), AP-1 (3.4e-10), and FOXO4 (4.6e-10). The presence of FOXO binding motifs confirms the identification of FOXO4 by GeneCodis^11–13^. AP-1 complexes and transcription factors from the NFAT family have been reported to act synergistically on certain promoters containing adjacent binding sites^26^; however, the AP-1 regulated genes show a larger overlap with the LEF1 and FOXO4 down-regulated genes (Fig. 4D). Secondly, we used the RNA-seq dataset to compute correlation coefficients between the sum of the expression levels of all the consensus down-regulated genes and each of the genes coding transcription factors (see Table S3). The 8th most correlated transcription factor with a Spearman correlation coefficient of 0.72 and a p-value of 5.64e-8 was FOSL1 and the 10th, with a Spearman correlation coefficient of 0.66 and a p-value of 1.64e-6, was FOSL2, both being the components of the AP-1 complex. The AP-1 transcription factor is a dimeric complex formed by members of the FOS and JUN families. The observed correlations suggest that a lower expression of AP-1 complex components in cancer cell lines is indeed responsible for the lower expression of the consensus down-regulated genes. The RNA-seq data further confirmed that the genes coding 6 different AP-1 components are strongly down-regulated in cancer cell lines (Table S4), in some cases with expression levels one hundred times lower in cancer cell lines compared to mesenchymal stem cells. As an illustration of this phenomenon, the expression level of the FOS transcription factor (the main member of the FOS family) was plotted versus the expression level of the consensus down-regulated genes (Fig. 4E).

The role of AP-1 in tumorigenesis has been described to be ambivalent^29^. Some of the components of the AP-1 complex have been reported to be pro-oncogenic or anti-oncogenic depending on tumor type, stage and genetic background. However, the gene expression data that have been analyzed here show a very clear down-regulation of at least 6 AP-1 components in 44 cancer cell lines compared to mesenchymal stem cells. An enrichment analysis using DAVID 6.7^25, 26^ revealed that 119 of the consensus down-regulated genes are membrane proteins and extracellular proteins, many of them being involved in cell attachment, such as fibronectin, thrombospondin, collagen, etc. (see Supplementary File SF3). It is known that cancer cells are less adhesive than normal human endothelial cells and fabricate less extracellular matrix, which favors their mobility and metastasis^30^. It is therefore possible that the down-regulation of AP-1 promotes cell migration and metastasis while at the same time could have anti-oncogenic effects in other stages of tumor development.

### Transcriptional basis of cancer hallmark capabilities

In order to determine the relationship between the two large-scale transcriptional patterns (epithelial-mesenchymal transition and AP-1 down-regulation) that appear to differentiate cancer cell lines from healthy dividing cells and the hallmark capabilities of cancer^9^, we performed enrichment analysis of KEGG^31, 32^ pathways using DAVID 6.7^25, 26^ (Supplementary file SF3) for the gene sets regulated by SP1, LEF1, and FOXO4 as well as for the consensus down-regulated genes and consensus up-regulated genes (found in both microarray and RNA-seq analysis). The mentioned analysis allowed us to identify the hallmark capabilities related to epithelial-mesenchymal transition and AP-1 down-regulation, respectively.

*Hallmark capabilities enabled by epithelial-mesenchymal transition* include *sustained proliferation*, with cell cycle components controlled by SP1, LEF1, and FOXO4 up-regulated in cancer cell lines. LEF1 also regulates the actin cytoskeleton, which is related to the formation of invasive protrusions involved in cell *invasion and metastasis* ^30^.

#### Hallmark capabilities enabled by putatively AP-1 and NFAT mediated down-regulation

Among the consensus down-regulated genes, KEGG pathway enrichment analysis revealed ECM-receptor interaction and focal adhesion, which are the features related to cell *invasion and metastasis* ^30^. Loss of expression of cytokine-cytokine receptors is related to both *evading growth suppressors* and *resisting cell death*, as illustrated, for example, by the loss of the pro-apoptotic receptor Fas receptor, which triggers the extrinsic apoptotic program. In general, this down-regulation pattern results in the loss by cancer cells of their ability to integrate environmental signals of different types.

### Hallmark capabilities enabled by alternative mechanisms

Fig. 2A–C shows that the up-regulation pattern associated to epithelial-mesenchymal transition is more pronounced in cancer cell lines than in mesenchymal stem cells. An enrichment analysis performed with DAVID 6.7 on the consensus up-regulated genes revealed the transcription factors E2F (p-value 1.4e-3) and NFY (p-value 1.4e-2). E2F is known to regulate the cell cycle, and the functional enrichment analysis of the consensus up-regulated genes revealed 10 components of the cell cycle to be up-regulated in cancer cell lines with respect to mesenchymal stem cells (Supplementary File SF3). Other enriched KEGG pathways among the consensus up-regulated genes were purine and pyrimidine metabolism, which are consistent with higher proliferation rates and an increased demand of biomass building blocks.

### Alterations in metabolism as an emerging hallmark of cancer

The well-known review by Hannahan and Weinberg^9^ indicates reprogramming energy metabolism as an emerging hallmark of cancer, which they describe as the shift to aerobic glycolysis or Warburg effect. Many cancer-specific metabolic features beyond the Warburg effect have been described in recent years^33^. Using a genome-scale metabolic model^34^ and an algorithm based on flux balance analysis (supplementary material), a search for metabolic sub-networks associated with the identified transcriptional patterns was performed.

It was observed that LEF1 up-regulates 4 different histone lysine methylases: EHMT2 and EHMT1, which methylate lysine 9, as well as EZH2 and NSD2, which methylate lysine 27. Lysine 27 histone trimethylation has been reported to be responsible for gene silencing in cancer^35^. An enrichment test of genes repressed by this mechanism and the consensus down-regulated genes was performed, but no significant enrichment was detected (data now shown).

**Figure 5:**
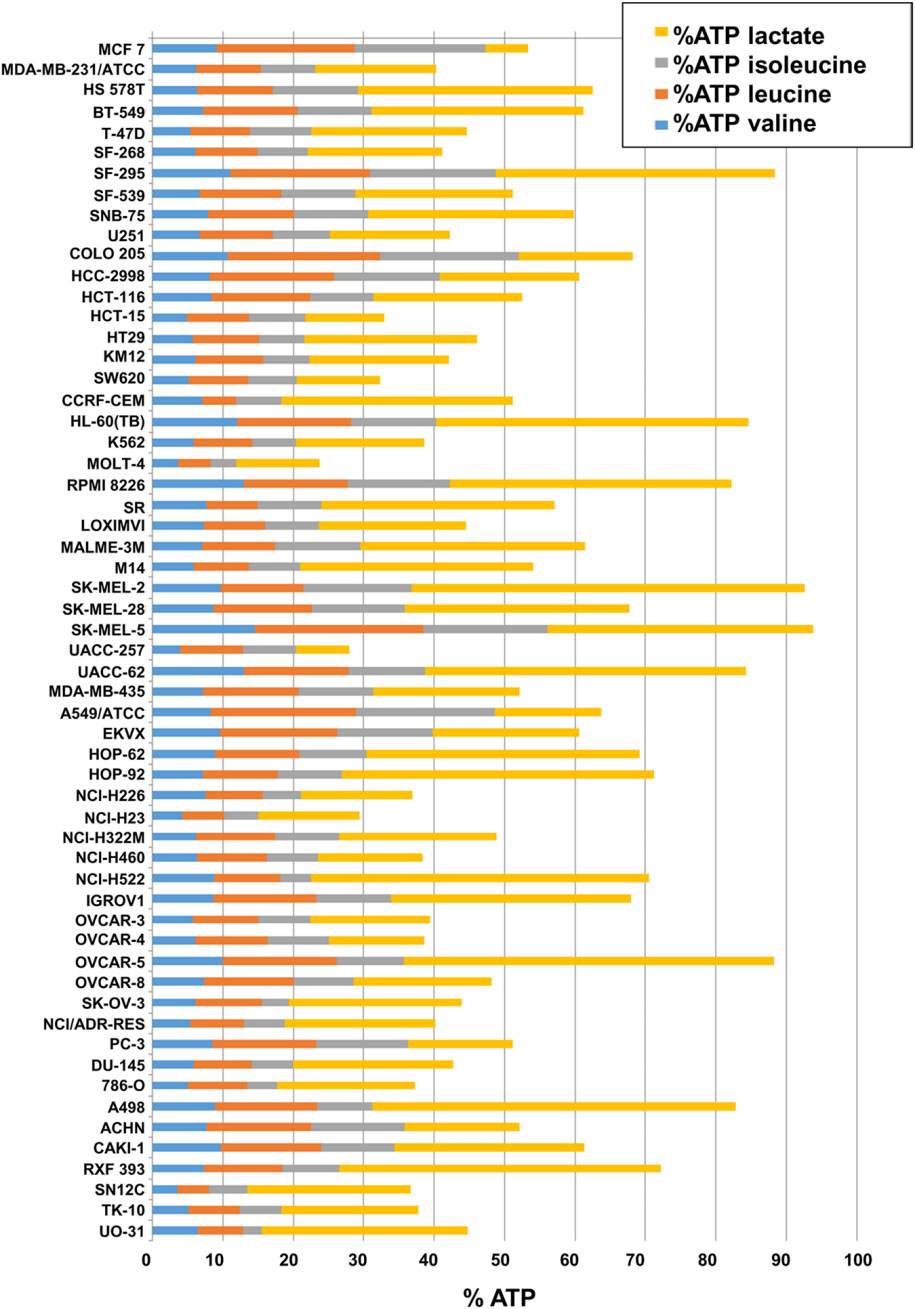
Estimated percentages of ATP obtained from lactic fermentation and valine, leucine, and isoleucine degradation for the NCI-60 cell lines.

More importantly, cancer cell lines differ from healthy dividing cells in the higher expression of 5 enzymes involved in valine degradation (BCAT2, DBT, DLT, HADHA, and HIBADH), with 4 of them (BCAT2, DBT, DLT, and HADHA) being also involved in isoleucine degradation and 3 in leucine degradation (BCAT2, DBT and DLT) (Supplementary Fig. S2). This suggests that the degradation of the mentioned branched chain amino acids could play an important role in the energy supply of cancer cells. In order to quantify this role, we used the mentioned genome-scale metabolic model together with experimental metabolite uptake rates of the NCI-60 cell lines^36, 37^. For each cell line, we computed a flux distribution satisfying the observed uptake and secretion rates and minimizing the sum of all the metabolic fluxes. From the obtained flux distribution, we estimated the total ATP production (which is equal to its consumption according to the pseudo-steady state assumption). The experimental lactic acid production rates and consumption rates of each of the mentioned branched chain amino acid were used to determine ATP produced by lactic fermentation of glucose and full degradation and oxidation of leucine, isoleucine, and valine. The fractions of each of the 4 ATP sources were plotted for each cell line (Fig. 5). It was found that between 12% and 55% of the total ATP is generated by the degradation of branched chain amino acids.

The 5 mentioned enzymes are not directly regulated by LEF1, FOXO4, or SP1 (according to GeneCodis); however, their expression pattern is identical to that of the genes associated with epithelial-mesenchymal transition (Supplementary Fig. S3), which suggests that the degradation of branched chain amino acids is triggered by the regulatory cascade associated with epithelial-mesenchymal transition.

### Degradation of branched chain amino acids as a potential therapeutic window

**Figure 6:**
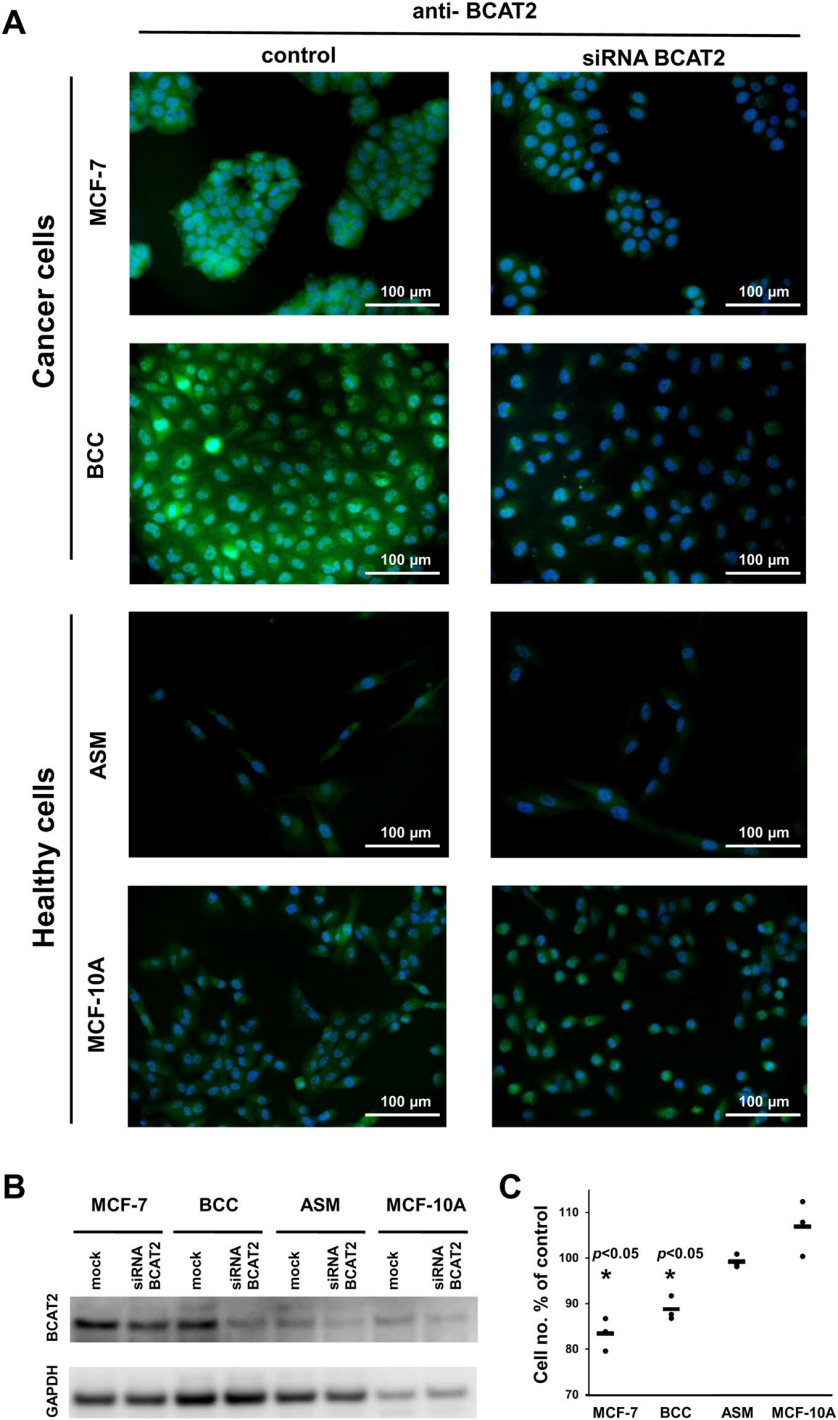
The effect of BCAT2 silencing on proliferation of breast cancer and normal cells. (**A**) Immunofluorescence of BCAT2 in cancer (MCF-7 and BCC) and normal (ASM and MCF-10A) cells in control and after BCAT2 siRNA transfection (green-secondary antibody to rabbit IgG-H&L conjugated with Alexa Fluor 488; blue-DAPI stained nucleus). (**B**) Representative Western blot showing BCAT2 expression levels after BCAT2 siRNA transfection compared to mock-treated controls. (**C**) Inhibition of cancer cell (MCF-7 and BCC) proliferation after BCAT2 silencing determined by flow cytometry assay. Data are expressed as a percentage of mock-treated controls (n = 3). All experiments were performed 24 h post-transfection.

The high fraction of ATP derived from the degradation of branched chain amino acids in cancer cell lines and its higher activity compared to some healthy dividing cells suggests that suppressing the activity of enzymes in this pathway could be a valid therapeutic strategy (alone or in combination with other treatments). Here we focused on the BCAT2 enzyme that catalyzed the first step of the degradation of leucine, valine, and isoleucine (Supplementary Fig. S4). Firstly, we confirmed at the protein level the conclusion obtained from microarray analysis. We used the breast cancer cell line MCF-7 and primary cell culture prepared from breast tumor tissue (BCC) (see Materials and Methods). We introduced BCC in our experimental design in order to determine whether the conclusions obtained in widely used cancer cell lines can be extrapolated to cells from real tumors. As healthy dividing cells, we chose airway smooth muscle cell line (ASM) and breast epithelial cell line (MCF-10A). As shown in Fig. 6A,B, higher abundance of BCAT2 in cancer cells was observed as compared with healthy cells.

BCAT2 gene silencing experiments in cancer and normal cells were performed to assess the impact of branched chain amino acid degradation on cell proliferation. Reduced levels of the target protein after siRNA transfection were evaluated by Western blotting and immunocytochemical analysis (Fig. 6A,B; for full images of Western blots see Supplementary Fig. S6). BCAT2 siRNA decreased the number of MCF-7 and BCC cells by 16.6% ± 2.1% and 11.3% ± 1.5%, respectively (Fig. 6C). The number of ASM and MCF-10A remained unchanged (99% ± 0.8% and 106% ± 3.4%, respectively, compared to control).

There was no change in viability of all tested cancer and healthy BCAT2 siRNA-treated cells, as determined by Annexin V-PE/7-AAD staining (Supplementary Fig. S5). These results suggest that BCAT2 could be a suitable drug target with cytostatic effects on cancer cells.

## Discussion

By analyzing 289 microarray samples and 50 RNA-seq libraries of cancer cell lines and healthy dividing cells, we have observed that cancer cell lines are generally characterized by two distinct regulatory events involving hundreds of genes.

The first identified event is associated with the phenomenon of epithelial-mesenchymal transition and appears to be more intense in cancer cell lines and healthy mesenchymal stem cells compared to epithelial stem cells. Based on gene enrichment analysis, the transcription factors SP1, LEF1, and FOXO4 have been identified as putative drivers of this large-scale transcriptional pattern. SP1 is already known to be involved in epithelial-mesenchymal transition, and our analysis revealed that among the genes up-regulated by SP1, there are 9 components of the Wnt signaling pathway, whose activation results in the phosphorylation of β-catenin, which binds the transcription factors coded by LEF1 and FOXO4, leading to the up-regulation of their target genes. The SP1 gene is itself among the FOXO4 targets and was found to be up-regulated in cancer cell lines. This suggests the existence of a positive activation feedback involving SP1, the canonical Wnt signaling pathway and FOXO4, resulting in a large-scale transcriptional change. It has been shown^38^ that switches between two alternative cellular states (such as epithelial and mesenchymal) are typically regulated by sets of transcription factors forming feedback structures.

Interestingly, 5 enzymes involved in the degradation of branched chain amino acids (leucine, valine, and isoleucine) show a transcriptional pattern associated with epithelial-mesenchymal transition, even if they are not directly regulated by SP1, LEF1, and FOXO4. This general observation at the gene expression level was confirmed at the protein level for the enzyme BCAT2 in the cancer cell line MCF-7, the primary cell culture BCC isolated from tumor (which showed high BCAT2 levels), ASM and MCF-10A cells, in which it was lowly expressed. The silencing of the BCAT2 gene led to a significant decrease of cell proliferation for MCF-7 and BCC while had no effect on the proliferation of ASM cells, which proved the existence of a therapeutic window that could be exploited to develop cytostatic drugs targeting CICs preferentially while having lower effects on healthy dividing cells.

A second large-scale transcriptional pattern appeared to differentiate cancer cell lines from healthy mesenchymal stem cells. This pattern consists on the apparent failure of cancer cell lines to overexpress a large number of genes coding membrane proteins involved in phenomena such as ECM-receptor interaction, focal adhesion, cytokine-cytokine receptors, etc., as well as extracellular proteins that constitute the extracellular matrix. This down-regulation appears to confer the cells with the abilities of evading growth suppressors and cell death and diminishing cell adhesion, which leads to invasion and metastasis. In healthy cells, these genes appear to be up-regulated as a result of epithelial-mesenchymal transition; however, they seem to avoid overexpression in cancer cell lines. A gene enrichment analysis as well as a correlation analysis between the expression of the genes down-regulated in cancer cell lines and the expression of each transcription factor (using RNA-seq data) revealed a putative role of the AP-1 transcriptional complex on the observed regulatory pattern. Among the genes coding proteins that form the AP-1 complex, 6 of them also appeared to be down-regulated in cancer cell lines with respect to healthy mesenchymal stem cells. The role of AP-1 in cancer development is controversial and some of the components of the AP-1 complex have been reported to be pro-oncogenic or anti-oncogenic depending on tumor type, stage, and genetic background; however, our analysis revealed strong evidence of low AP-1 activity in cancer cell lines. Therefore, we believe that the AP-1 transcriptional complex deserves further attention for understanding the mechanisms leading to tumor formation and metastasis.

Our experiments on BCAT2 demonstrate how the described transcriptional hallmarks could potentially be exploited as therapeutic windows to target selectively cancer cells while keeping unaffected healthy dividing cells.

## Materials and Methods

### Microarrays

To extract the characteristic direction in gene expression that differentiates tumorigenic and healthy duplicating cells, we used the data in Table S1. The microarray set in Table S1 was normalized using the RMA method implemented in the *affy* R package.

Linear discrimination analysis and identification of differentially expressed genes was performed as described in the supplementary material (Statistical methods). The R code used in the analysis is reported in the supplementary material (R code to perform linear discrimination analysis).

As validation sets, we used the data summarized in Table S2. We used two validation datasets that included two different cancer cell lines originated in the same tissue (colon and breast, respectively) and compared them with stem cells from the same tissue.

Each validation set (colon and breast respectively) was normalized using the RMA method implemented in the *affy* R package.

### RNA-seq analysis

Gene expression data for the 44 cancer cell lines (BioProject accession number PRJNA183192) included in the Human Proteome Atlas were downloaded from www.proteinatlas.com in the form of a comma-separated file that contains the expression of each gene in each cell line (given as RMPK). This file was parsed using a customized python script (available upon request). RNA-seq fastq files for 6 different mesenchymal stem cell lines (3 from placenta and 3 from bone-marrow) were downloaded from https://usegalaxy.org/u/cic19/h/mesenchymal-stem-cells-rnaseq. The reads were aligned on the complete list of human transcripts obtained from Ensembl BioMart using Bowtie2. The resulting alignment files were analyzed using a customized python script that is available upon request. Fold changes were calculated by dividing the average expression of each gene in cancer cells by the average expression in mesenchymal stem cells, p-values were calculated using the t-test, and correction for multiple testing was performed as described in the supplementary material.

### Cell Lines and Culture Medium

BCC cells were prepared as described elsewhere (https://www.ncbi.nlm.nih.gov/pmc/articles/PMC4063716/) from part of the tumor tissue sample taken from a 41-year-old female patient suffering from invasive ductal carcinoma (T1 N0 M0 G2, ER(+), PR(+), HER2(3+)) during lumpectomy and sentinel lymph node biopsy surgery. The second part of the sample was used for histological and immunohistochemical examination. Invasive ductal carcinoma staging (T (tumor), N (node), M (metastasis) and G (grade)) categories were assigned according to the Union for International Cancer Control (UICC) classification. Pathohistological diagnosis was proved at the Department of Pathology, Lithuanian University of Health Sciences. The patient provided written informed consent. This study was approved by the Kaunas Regional Biomedical Research Ethics Committee (license number BE-2–7), and all experiments were performed in accordance with the guidelines and regulations of the mentioned institution. BCC and MCF-7 (human breast adenocarcinoma cell line; CLS-Cell Lines Service, Eppelheim, Germany) were grown in Dulbecco’s Modified Eagle Medium:Ham’s F-12 (1:1; DMEM/F-12) (Life technologies, Carlsbad, CA, USA) medium with 10% fetal bovine serum (FBS) and antibiotics (penicillin 100 U/mL, streptomycin 100 µg/mL). Immortalized human airway smooth muscle (ASM) cells (kindly donated by Prof. R. Gosens, University of Groningen, Netherlands) were obtained as described elsewhere^39^. ASM cells were grown in DMEM medium containing 10% FBS and penicillin/streptomycin mix (100 U/mL penicillin and 100 μg/mL streptomycin). MCF-10A cells (ATCC, Wesel, Germany) were grown in DMEM/F-12 medium supplemented with 5% horse serum (GE Healthcare Life Sciences, Logan, USA), 20 ng/mL EGF (Life technologies, Carlsbad, CA, USA),10 μg/ml insulin (Life technologies, Carlsbad, CA, USA), 0.5 μg/mL hydrocortisone, and 100 ng/mL cholera toxin and penicillin/streptomycin mix. Cells were maintained in a humidified incubator at 37 °C/5% CO_2_. All chemicals were purchased from Sigma Aldrich Corp. (Steinheim, Germany), unless indicated otherwise.

### siRNA transfection

For BCAT2 gene silencing, jetPRIME transfection reagent (Polyplus-transfection® SA, France) and final concentration of 5 nM BCAT2 siRNA (sense 5′-CCAUGAACAUCUUUGUCUAtt-3′, antisense 5′-UAGACAAAGAUGUUCAUGGtt-3′, Invitrogen, USA) were used according to the manufacturer’s instructions. All the following experiments were performed 24 h after siRNA transfection.

### Immunocytochemistry

Cells grown in 24-well plates with glass coverslips on the bottom were fixed with 4% paraformaldehyde for 15 min and permeabilized with 0.2% Triton X-100 in PBS for 3 min. Samples then were incubated with primary antibody against BCAT2 (Abcam, Cambridge, UK) for 1 h at 37 °C, then rinsed with 1% BSA/PBS and incubated with secondary donkey anti-rabbit IgG H&L antibody conjugated with Alexa Fluor 488 (Life technologies, Carlsbad, CA, USA) for 30 min. Coverslips were attached with a Vectashield Mounting Medium with DAPI (Vector Laboratories, CA, USA). The analysis was performed with an inverted fluorescence microscope Olympus IX81 (Olympus Europa holding Gmbh, Hamburg, Germany) equipped with an Orca-R2 cooled digital camera (Hamamatsu Photonics K.K., Japan), the fluorescence excitation system MT10 (Olympus Life Science Europa Gmbh, Hamburg, Germany), and the fluorescence imaging system XCELLENCE (Olympus Soft Imaging Solutions Gmbh, München, Germany).

### Western Blot analysis

For each cell type, 3 × 10^6^ cells were lysed in ice-cold cell extraction buffer (Invitrogen, USA) containing 1 mM PMSF (Abcam, Cambridge, UK) and 20 μl/mL protease inhibitor cocktail (Sigma-Aldrich, Steinheim, Germany) for 30 min. The lysates were centrifuged at 13000 rpm for 10 min at 4 °C. The total protein concentration was determined by a Qubit® protein assay kit (Invitrogen, USA) using a Qubit 3.0 fluorometer (Invitrogen, USA). Cell lysates (30 μg) were separated by Bolt™ 4–12% Bis-Tris plus gels (Invitrogen, USA) and transferred to PVDF membranes (Millipore, USA). Proteins were detected using primary antibodies against BCAT2 (Abcam, Cambridge, UK) and GAPDH (Invitrogen, USA) and a WesternBreeze® chemiluminescent kit (Invitrogen, USA) according to the manufacturer’s instructions. Bands were visualized using the G:Box Chemi Gel Documentation system (Syngene, USA).

### Flow cytometry assay

Initially, 5 × 10^4^ cells were seeded in a 6-well plate in 2 mL of full media. Mock-transfected and BCAT2 siRNA-transfected cells were washed twice with PBS, trypsinized, and collected by centrifugation. Afterwards, cells were incubated with Guava Nexin® Reagent (Millipore, USA) for 20 min at room temperature according to the manufacturer’s instructions. Samples were quantified by a Guava PCA flow cytometer (Millipore, USA). The data were analyzed by guavaSoft 2.7 Nexin software.

### Statistical analysis

Averaged experimental results are reported as means ± standard error of the mean. Statistical analysis was performed using two-tailed Student’s t-test. Differences were considered statistically significant at p < 0.05. The calculations were performed using SigmaPlot.

## Electronic supplementary material

Supplementary Material

Supplementary file 2

Supplementary file 3
