# Supplementary material for: Transcriptional hallmarks of cancer cell lines reveal an emerging role of branched chain amino acid catabolism
Source: Sci Rep. 2017 Aug 10;7:7820. doi: 10.1038/s41598-017-08329-8 (PMC5552680; doi:10.1038/s41598-017-08329-8)
Supplement: Supplementary file 1 — Supplementary Material [file 41598_2017_8329_MOESM1_ESM.pdf]

Supplementary material for the article “Transcriptional hallmarks of cancer cell lines reveal an emerging role of branched chain amino acid catabolism”

Ieva Antanavičiūtė[1], Valeryia Mikalayeva[1], Ieva Ceslevičienė[1], Gintarė Milašiūtė[1], Vytenis Arvydas Skeberdis[1], Sergio Bordel[1,2]

1 Institute of Cardiology, Lithuanian University of Health Sciences, 15 Sukilėlių Ave., LT- 50162, Kaunas, Lithuania
2 Department of Biology and Biological Engineering, Chalmers University of Technology, Kemivägen 10, SE-41296, Göteborg, Sweden.

Content:

- **Statistical methods**
- **R code to perform linear discrimination analysis**
- **Computations using genome scale metabolic models**
- **Supplementary tables**
- **Supplementary figures**

Statistical methods

Linear discrimination analysis:

The normalized expression profiles obtained from each micro-array are vectors with as many components as probes in the microarray. The analyzed samples can be classified into classes, which in our case are CICs and healthy dividing cells. Within each class, the expression profile is assumed to follow a multivariate Gaussian probability distribution.

$$f_k(\vec{x}) = \frac{1}{(2\pi)^{\frac{p}{2}} |\Sigma|^{\frac{1}{2}}} \exp\left(-\frac{1}{2} (\vec{x} - \vec{\mu}_k)^T \Sigma^{-1} (\vec{x} - \vec{\mu}_k) \right) \quad (1)$$

We assume the covariance matrix Σ to be the same for each class. The parameter *p* indicates the number of variables (probes in the microarray).

This density function can be seen as the conditional probability of $\vec{x}$ conditioned to the class being *k*. Using the Bayes' theorem we can obtain the probability of a sample belonging to the class *k* conditioned to its expression profile being $\vec{x}$.

$$P(k|\vec{x}) = \frac{f_k(\vec{x}) P(k)}{P(\vec{x})} \quad (2)$$

If we want to discriminate between two classes (1 and 2) by observing the expression profile $\vec{x}$ we can compute the log-ratio of their respective conditioned probabilities:

$$\log \frac{P(2|\vec{x})}{P(1|\vec{x})} = \log \frac{P(2)}{P(1)} + \log \frac{f_2(\vec{x})}{f_1(\vec{x})} \quad (3)$$

By using the multivariate Gaussian model described in equation (1) we observe that the log-ratio follows has a linear dependence on the observed $\vec{x}$.

$$\log \frac{P(2|\vec{x})}{P(1|\vec{x})} = \log \frac{P(2)}{P(1)} + \frac{1}{2} (\vec{\mu}_2 - \vec{\mu}_1)^T \Sigma^{-1} (\vec{\mu}_2 - \vec{\mu}_1) + \frac{1}{2} (\vec{x})^T \Sigma^{-1} (\vec{\mu}_2 - \vec{\mu}_1) \quad (4)$$

The direction in the gene expression space in which the log-ratio grows faster is perpendicular to the hyper-plane that separates best both classes and is equal to:

$$\vec{d} = \Sigma^{-1} (\vec{\mu}_2 - \vec{\mu}_1) \quad (5)$$

In order to apply directly equation (5) we should first estimate the covariance matrix and compute its inverse. In our case, as we have more variables than samples, the covariance matrix computed from these samples will not be full rank and cannot be inverted.

Here we solve this problem by diagonalizing Σ and projecting $\vec{\mu}_2 - \vec{\mu}_1$ on the sub-space defined by its eigenvectors with non-zero eigenvalues. By taking just the non-zero eigenvalues, we obtain a full rank diagonal matrix that can be inverted just by taking the inverse of each eigenvalue. Multiplying this inverted matrix by the components of the projection of $\vec{\mu}_2 - \vec{\mu}_1$ on the space of eigenvectors with non-zero eigenvalues, we obtain

the direction of maximal increase of the log-ratio, within the mentioned sub-space (expressed using the base formed by the non-zero eigenvalues). In order to get a vector in the space expression profiles, it is enough to multiply each eigenvector by its component and sum the values. We then normalize this final vector in order to have a modulus equal to 1.

We have implemented these operations in an R script (provided in the supplementary material) that takes as an input a normalized expression set and the two classes to be compared. It returns the characteristic direction and a plot showing the positions of the projections of each sample along the characteristic direction. This allows visualizing how well the samples can be assigned to each class based on their expression levels. If the expression profile of a sample is given by the vector $\vec{x}$, its projection on the characteristic direction is just obtained as follows:

$$\Pr\left(\vec{x},\vec{d}\right) = \vec{x} * \vec{d} = \sum_i x_i d_i \qquad (6)$$

As the characteristic direction is defined to be a unitary vector, no normalization is needed.

Contribution of a gene (or probe) set to the characteristic direction:

In order to compute the contribution of a certain set of genes G to the value defined in equation (6), we define the projection of the characteristic direction on the considered gene set, by restricting the summation in equation (6) to the probes corresponding to the genes in G.

$$\Pr_G = \sum_{i \in G} x_i d_i \qquad (7)$$

If the expression levels of the genes in two sets G and F are independent variables, $\Pr_G$ and $\Pr_F$ will be normally distributed (according to the central limit theorem if the numbers of genes in each set are large enough) and will be independent from each other. The existence of correlations between $\Pr_G$ and $\Pr_F$ provide valuable information about common regulatory mechanisms of the genes in G and F.

For the gene-sets that are downregulated (negative contributions to the characteristic direction) we use the absolute values of the projection defined in equation (7).

Statistical test to quantify the significance of the contribution of each variable to the characteristic direction:

A new statistical test has been developed. It is based on the probability distribution of the co-sinus of the angle between a random vector in an n-dimensional Euclidian space and a certain sub-space P of m-dimensions. The co-sinus of the angle between two vectors in a Euclidian space is defined as follows:

$$\cos\theta = \frac{\vec{x} * \vec{y}}{|\vec{x}||\vec{y}|} \qquad (8)$$

In our case, the vector $\vec{y}$ is defined to be the projection of $\vec{x}$ on a certain sub-space, therefore the equation can be simplified as:

$$\cos\theta = \frac{\vec{y}^2}{|\vec{x}||\vec{y}|} = \frac{|\vec{y}|}{|\vec{x}|} \qquad (9)$$

If we choose to represent the vectors using an orthonormal basis so that m of its components are also a basis of the considered m-dimensional sub-space, we can rewrite equation (7) in function of the vector components:

$$\cos^2\theta = \frac{\sum_{i\in P} x_i^2}{\sum_i x_i^2} \tag{10}$$

The summation in the numerator is extended to the components that correspond to the sub-space P on which the vector $\vec{x}$ is being projected. The denominator contains all the components. The denominator can be decomposed into the contribution of the components in P and the contribution of the components in the sub-space orthogonal to P.

$$\cos^2\theta = \frac{\sum_{i\in P} x_i^2}{\sum_{i\in P} x_i^2 + \sum_{i\in \bar{P}} x_i^2} = \left(1 + \frac{\sum_{i\in \bar{P}} x_i^2}{\sum_{i\in P} x_i^2}\right)^{-1} \tag{11}$$

We want the vector $\vec{x}$ to have a random direction. A way to achieve it is to choose its component from normal distributions with zero average and standard deviation 1. In this case the summations that appear in equation (9) will follow χ^2 distributions:

$$\sum_{i\in P} x_i^2 \approx \chi_m^2 \tag{11}$$

$$\sum_{i\in \bar{P}} x_i^2 \approx \chi_{n-m}^2 \tag{13}$$

The ratio between them will have the following F probability distribution:

$$\frac{m}{n-m}\frac{\sum_{i\in \bar{P}} x_i^2}{\sum_{i\in P} x_i^2} \approx F_{n-m,m} \tag{14}$$

If we rewrite it in function of the co-sinus we obtain the following expression, which will allow us to compute p-values for single variables or for sets of variables:

$$\frac{m}{n-m}\left(\frac{1}{\cos^2\theta} - 1\right) \approx F_{n-m,m} \tag{15}$$

If we want to test the contribution of each individual probe to the characteristic direction, the sub-space P is just the corresponding probe and m=1. On the same way we can test the contribution of any desired probe-set.

Following this method, we have computed p-values for each probe in the hgu133 plus2 chip and corrected for multiple testing. The genes mapped to proves with false discovery rates lower than 0.05 have been considered as contributing significantly to the difference between CICs and healthy dividing cells (supplementary file S2). The calculations have been performed using a customized R script (provided in the supplementary material).

R code to perform linear discrimination analysis

This function takes as an input a normalized expression set and two groups of samples within the set (based on a particular label such as malignant or non-malignant). The function returns the characteristic direction, a p-value that quantifies the statistical significance of the separation of both groups along the characteristic direction, and a strip chart in which this separation is visualized.

```
#Calculates the characteristic direction of two data groups
#Takes as an imput an affy expression set and the lables of the two groups to be compared, group 2 is the reference
#Outputs a column vector with the characteristic direction

cd=function(expresionset,label,type1,type2){
                set1=expresionset[,expresionset[[label]]==type1]
                set2=expresionset[,expresionset[[label]]==type2]
                m1=rowMeans(exprs(set1))
                m2=rowMeans(exprs(set2))
                d=m1-m2
                n1=exprs(set1)-m1
                n2=exprs(set2)-m2
                n=cbind(n1,n2)
                pc=prcomp(t(n))
                for (i in 1:length(pc$sdev)){if (pc$sdev[i]<0.01*pc$sdev[1]){break}}
                l=i-1
                pcs=pc$rotation[,1:l]
                comps=t(d)%*%pcs
                w=c()
                for (i in 1:length(comps))
                                {peso=comps[i]/pc$sdev[i]
                                w=c(w,peso)}

                chd=w%*%t(pcs)
                chd=chd/sqrt(sum(chd^2))
                val1=chd%*%exprs(set1)
                val2=chd%*%exprs(set2)
                test=t.test(val1,val2)
                pv=test$p.value
                result=list(chdir=chd,values1=val1,values2=val2,p.value=pv)
                stripchart(x,group.names=c(type1,type2),xlab="Characteristic direction",col=c("red","blue"),pch=19,at=c(1.25,1.75))
                return(result)
                }
```

The following R function takes the characteristic direction obtained using the previous function and quantifies the contribution of each probe as it is described in methods. It returns the lists of up and downregulated genes respectively, calculated as it is described in methods.

```
difsets=function(direction){
                    up=c()
                    down=c()
                    n=length(direction)
                    x=hgu133plus2ENSEMBL
                    probes=mappedkeys(x)
                    xx=as.list(x[probes])
                    a=direction^2
                    rango=rank(a)
                    for (i in 1:n){

                              pv=pf((1/a[i]-1)/(n-1),n-1,1,ncp=0)
                              if (pv*(n/rango[i])<0.05){
                                        if (direction[i]>0){

          up=c(up,xx[colnames(direction)[i]])
                                        }
                                        if (direction[i]<0){

          down=c(down,xx[colnames(direction)[i]])
                                        }
                                        }

                              }
                    finup=c()
                    for (name in names(up)){finup=c(finup,up[[name]])}
                    findown=c()
                    for (name in names(down)){findown=c(findown,down[[name]])}
                    sets=list(up=unique(finup),down=unique(findown))
                    return(sets)
                    }
```

Computations using Genome Scale Metabolic Models

Heuristic algorithm to identify metabolic sub-networks differentially used by cancer cell lines:

There are several ways to extract differentially expressed gene sub-networks from metabolic or other biological networks. A common approach is to consider reactions that share one or more metabolites as adjacent. This has several limitations, because metabolite sharing does not necessarily indicate that the metabolic fluxes in two reactions are correlated, especially if the shared metabolite is present in many metabolic reactions, which is the case of cofactors such as ATP or Acetyl-CoA but also metabolites such as pyruvate. A second approach that we used in a previous article is to consider reactions that are stoichimetrically coupled, such as those in linear pathways. Here we use an intermediate approach which uses the stoichiometric topology of the network (not only metabolite sharing) but does not require the fluxes between reactions to be coupled for all the feasible flux distributions.

This method computes so called elementary flux modes of the network (minimal sets of reactions able to operate in steady state). These elementary flux modes are chosen to have the following characteristics:

1. Only metabolites experimentally observed to be up-taken from the growth medium by cancer cell lines are up-taken.

2. The net production of a chosen metabolite in the network is fixed to one unit.

3. The reactions containing genes down (or up) regulated in CICs are used as little as possible.

4. The reactions containing genes up (or down) regulated in CICs are allowed to be used without any restriction.

In this way each computed elementary flux mode corresponds to the simplest flux distribution that produces a particular metabolite using as little as possible the down-regulated reactions and as much as possible the up-regulated reactions.

In order to perform these computations we have modified the *optimize* function implemented in the COBRA Toolbox by introducing 2 extra arguments. The first argument is a vector of weights for each reaction in the model. The reactions set to be used as little as possible have a weight of -100, the reactions set to be used as much as possible have a weight of 0, and the rest a weight of -1. The second argument is the index of one metabolite in the network. The net production of this metabolite is going to be set to 1. This modified function has been named *optimizeW* and is available upon request. The output of this function is the flux distribution that produces a unit of the specified metabolite and maximizes the sum of the fluxes in each reaction multiplied by the corresponding weights.

By solving the function for each metabolite in the model we obtain a set of elementary flux models. In order to identify metabolic sub-networks that are up-regulated (or down-regulated), it has been used a hypergeometric statistical test that compares the fraction of up-regulated metabolic genes associated to the elementary flux mode with the total fraction of upregulated metabolic genes in the whole metabolic network.

The most statistically significant elementary flux modes contain (within the reactions that have non-zero flux in these flux modes) differentially expressed reaction sub-networks that are stoichiometrically related to each other.

The described computations have been performed in MATLAB using the previously mentioned optimization function.

Supplementary tables:

GEO accession code	Description of the samples	Number of microarrays
GSE32474	NCI-60 cancer cell lines	174
GSE15543	Beta cells from pancreatic islets	33
GSE32719	Hematopoietic stem cells	27
GSE57255	Dental pulp stem cells	24
GSE12155	Endothelial progenitor cells	24
GSE48022	Mesenchymal stem cells	7

Table S1. *Microarrays used to identify the characteristic gene expression direction discriminating tumorigenic from healthy dividing cells.*

GEO accession code	Description of the samples	Number of microarrays
GSE11440	Colon cancer cell line HT29	6
GSE11618	Colon cancer cell line HCT116	18
GSE31255	Colon stem cells	11
GSE27515	Breast cancer cell line MDA231	3
GSE12155	Breast cancer cell line ZRT75	3

GSE48022	Mammary epithelial stem cells	3

Table S2. *Microarrays used to validate the obtained characteristic direction.*

Transcription factors whose expression is correlated with the consensus downregulated genes:

Ensembl gene ID	R	p-val
ENSG00000100644	0.751	6.55E-9
ENSG00000118513	-0.749	7.35E-9
ENSG00000125398	0.749	7.57E-9
ENSG00000187079	0.744	1.09E-8
ENSG00000141968	-0.736	1.81E-8
ENSG00000185811	-0.734	2.17E-8
ENSG00000175592	0.719	5.64E-8
ENSG00000149480	-0.676	6.54E-7
ENSG00000141384	-0.663	1.26E-6
ENSG00000075426	0.658	1.64E-6
ENSG00000168874	0.656	1.76E-6
ENSG00000164104	-0.651	2.28E-6
ENSG00000106948	-0.651	2.28E-6
ENSG00000095574	-0.643	3.35E-6
ENSG00000109381	-0.639	4.03E-6
ENSG00000149948	0.627	6.94E-6
ENSG00000072364	0.627	6.98E-6
ENSG00000112033	0.625	7.32E-6
ENSG00000037965	0.624	7.85E-6
ENSG00000168036	0.624	7.95E-6
ENSG00000170485	0.623	7.99E-6
ENSG00000161405	-0.620	9.40E-6

Table S3. *Spearman correlation coefficients and p-values between the aggregated expression level of the consensus downregulated genes (those found downregulated in both the microarray and RNA-seq analysis) and the expression levels of genes (Ensembl gene IDs) coding transcription factors (the 20 most correlated are shown).*

Gene	log10(FC)	p-value
FOS	-2.27	2.58E-17
FOSB	-2.13	7.48E-09
JUN	-0.775	5.58E-10
JUNB	-0.739	2.01E-07
FOSL1	-0.479	3.52E-03
FOSL2	-0.448	2.29E-02

Table S4. *Fold changes (decimal logarithms) and p-values resulting from comparing the expression of 6 components of the AP-1 complex between mesenchymal stem cells and cancer cell lines.*

Supplementary figures:

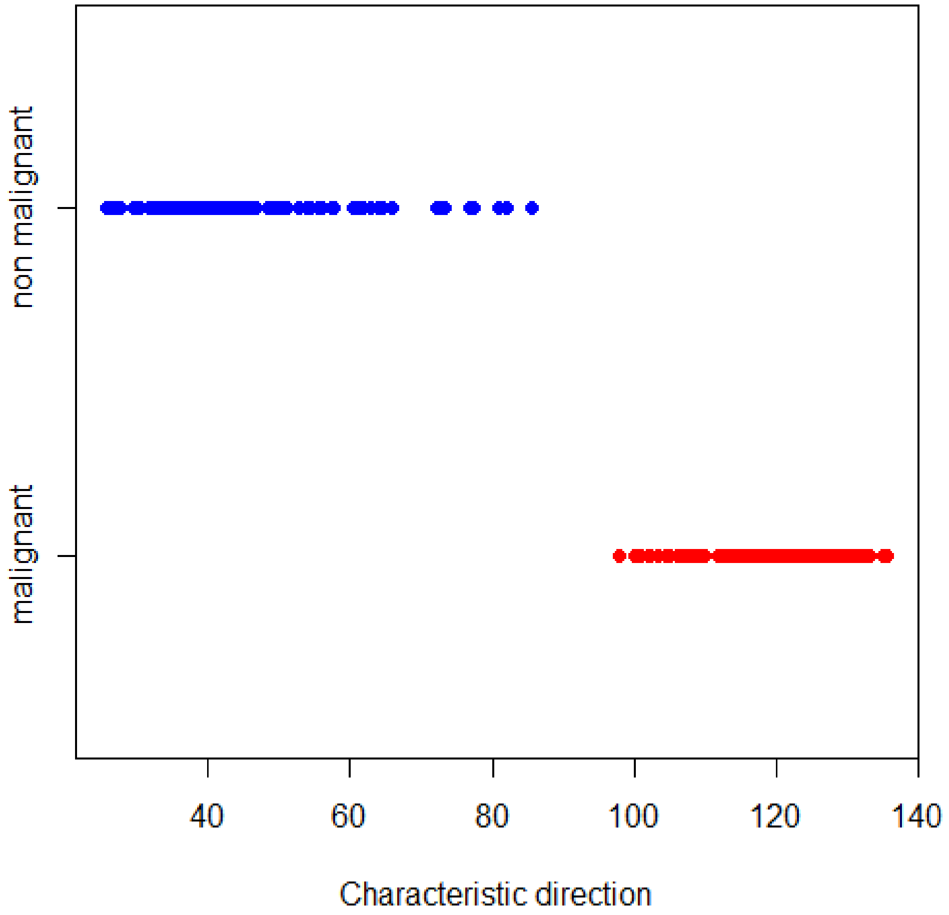

Figure S1. Projection of the expression profiles on the characteristic direction. We can see that tumorigenic dells can be fully discriminated from healthy dividing cells based on this projection.

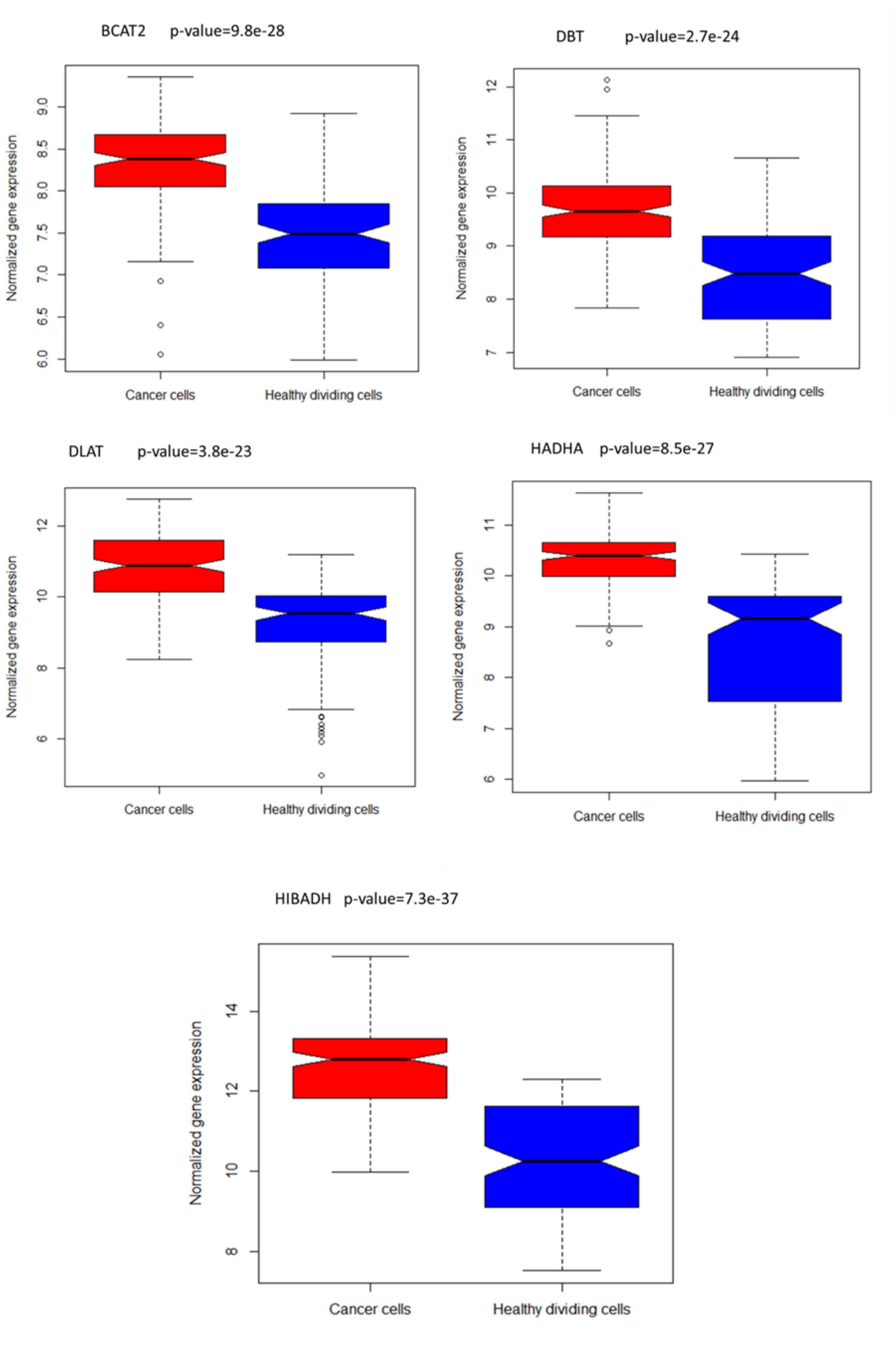

Figure S2. Box plots illustrating the differences of the expression of 5 metabolic genes involved in amino-acid catabolism in cancer cell lines and healthy dividing cells.

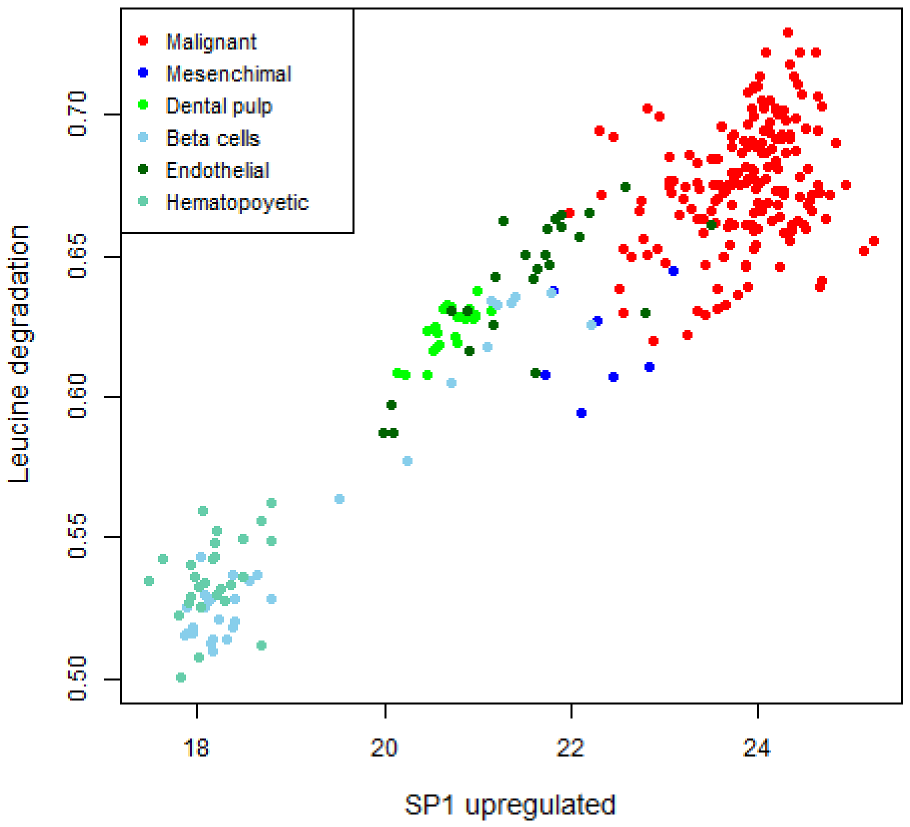

Figure S3. *Correlation between the aggregated expressions of SP1 upregulated genes and 5 genes involved in leucine, isoleucine and valine degradation (BCAT2, DBT, DLT, HADHA and HIBADH).*

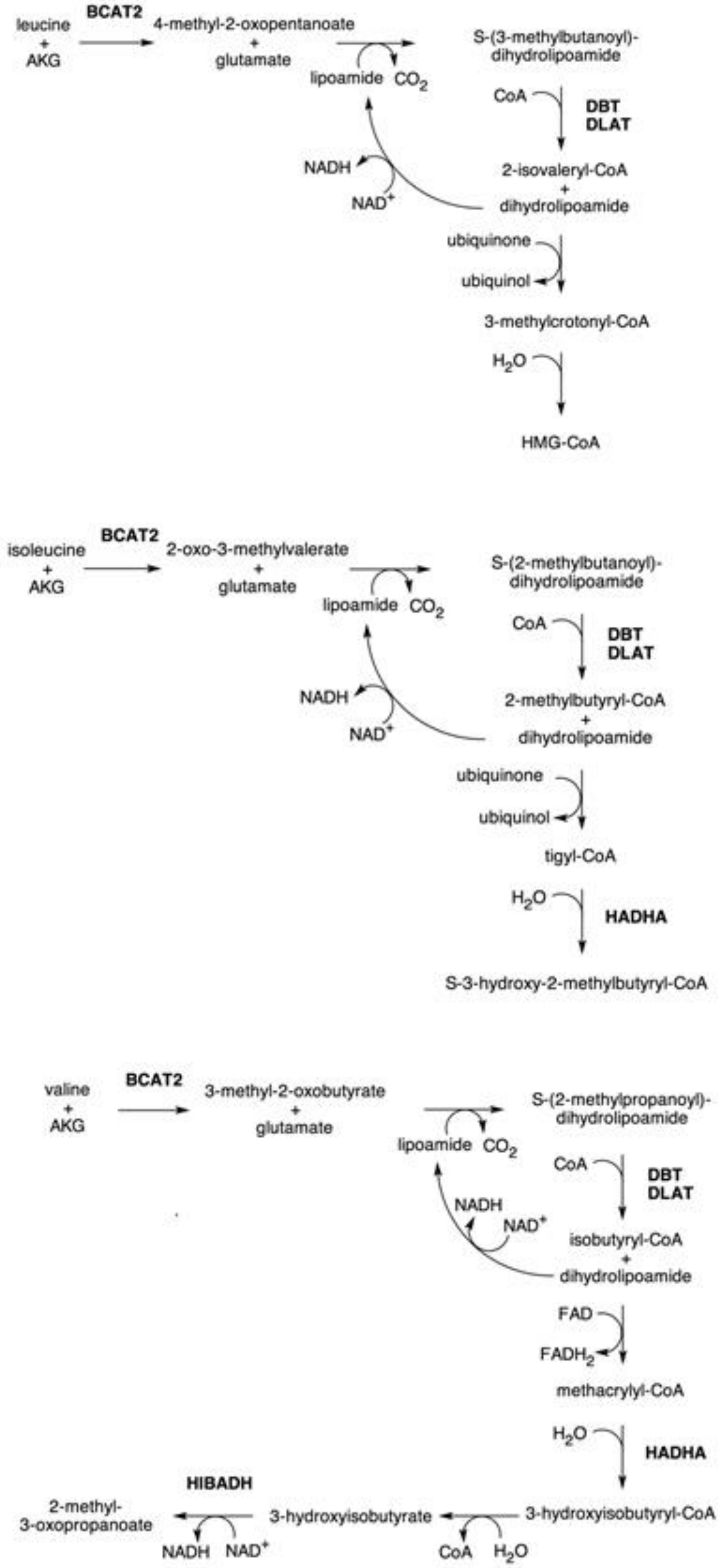

Figure S4. *Illustration of the, leucine, isoleucine and valine degradation pathways*

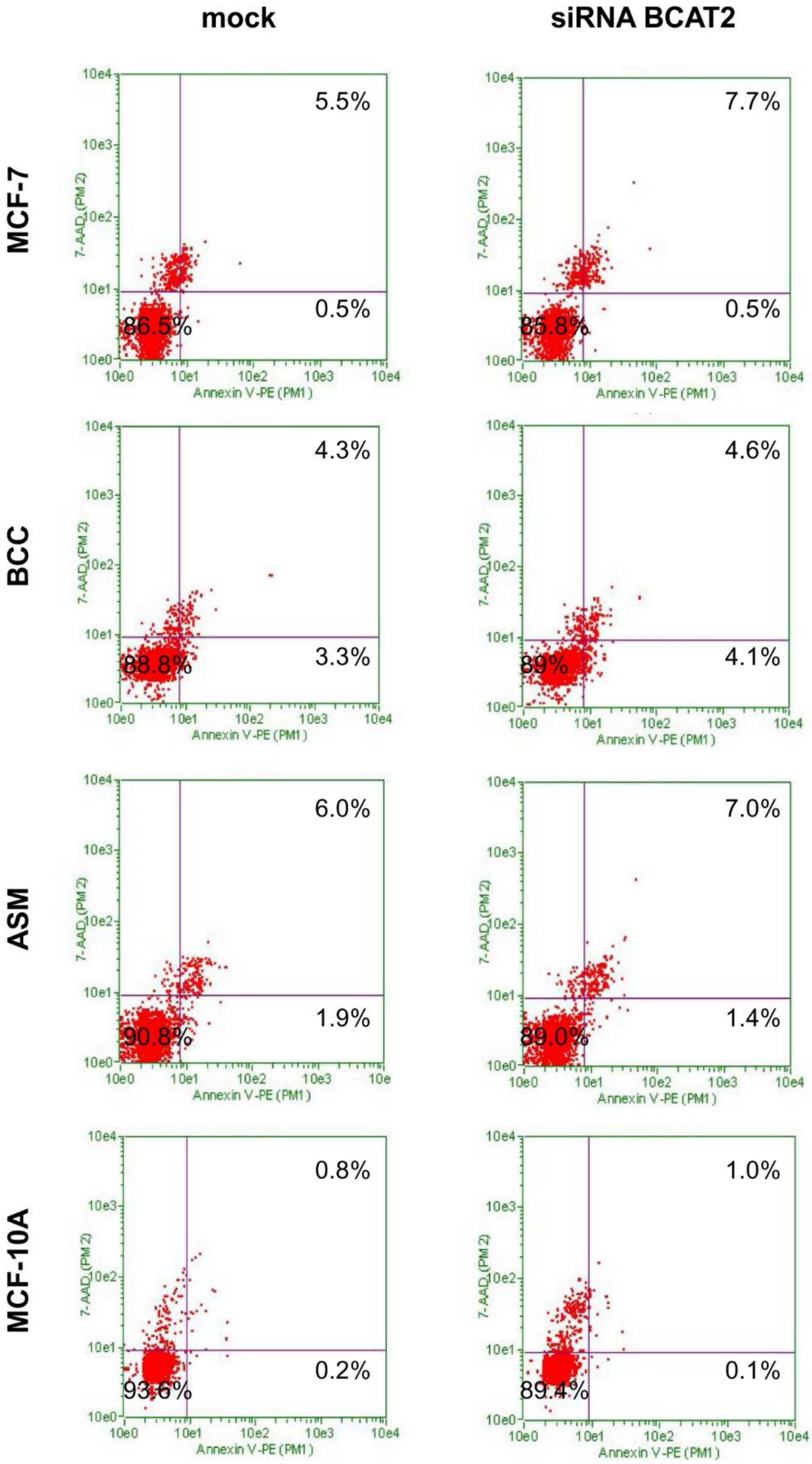

Figure S5. Representative images of flow cytometric analysis using Annexin V-PE/7-AAD staining. *The numbers presented in lower left panel of each graph indicate the amount of intact cells, in the lower right panels - early apoptotic cells,in the upper right panels - late apoptotic cells. Cell viability did not differ in control and BCAT2 siRNA treated cells.*

BCAT2

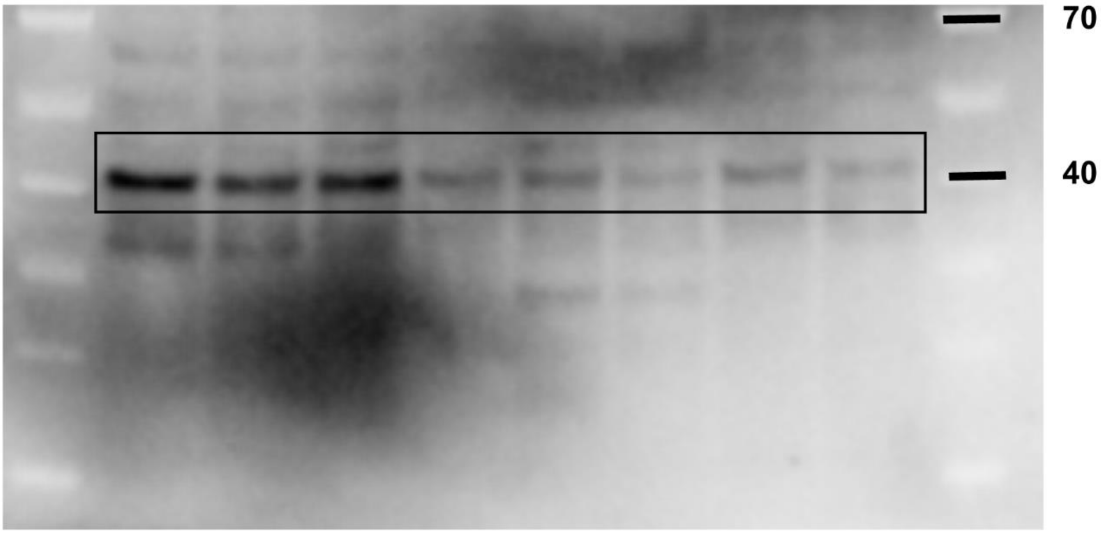

GAPDH

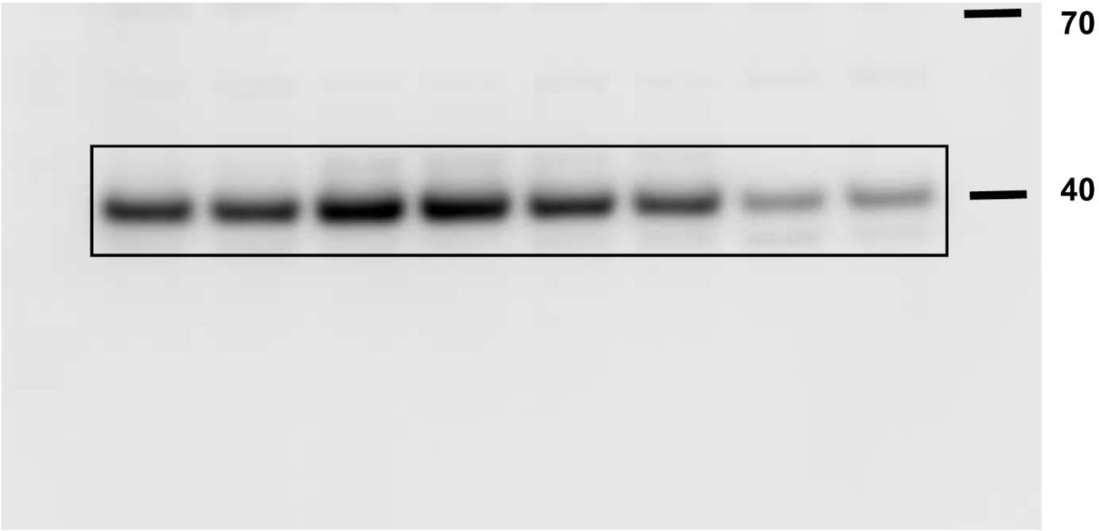

Figure S6. *Full images of Western blots.*
